# Identification of hypothermia-inducing neurons in the preoptic area and activation of them by isoflurane anesthesia and central injection of adenosine

**DOI:** 10.1186/s12576-024-00927-2

**Published:** 2024-06-12

**Authors:** Erika Uchino, Ikue Kusumoto-Yoshida, Hideki Kashiwadani, Yuichi Kanmura, Akira Matsunaga, Tomoyuki Kuwaki

**Affiliations:** 1 Department of Physiology, Graduate School of Medical and Dental Sciences, Kagoshima University, Sakuragaoka 8-35-1, Kagoshima, 890-8544 Japan; 2 Department of Anesthesiology, Graduate School of Medical and Dental Sciences, Kagoshima University, Kagoshima, Japan

**Keywords:** Torpor, Preoptic area, Hypothermia-inducing neurons, Anesthesia, Adenosine

## Abstract

Hibernation and torpor are not passive responses caused by external temperature drops and fasting but are active brain functions that lower body temperature. A population of neurons in the preoptic area was recently identified as such active torpor-regulating neurons. We hypothesized that the other hypothermia-inducing maneuvers would also activate these neurons. To test our hypothesis, we first refined the previous observations, examined the brain regions explicitly activated during the falling phase of body temperature using c-Fos expression, and confirmed the preoptic area. Next, we observed long-lasting hypothermia by reactivating torpor-tagged Gq-expressing neurons using the activity tagging and DREADD systems. Finally, we found that about 40–60% of torpor-tagged neurons were activated by succeeding isoflurane anesthesia and by icv administration of an adenosine A1 agonist. Isoflurane-induced and central adenosine-induced hypothermia is, at least in part, an active process mediated by the torpor-regulating neurons in the preoptic area.

**Graphical abstract:**

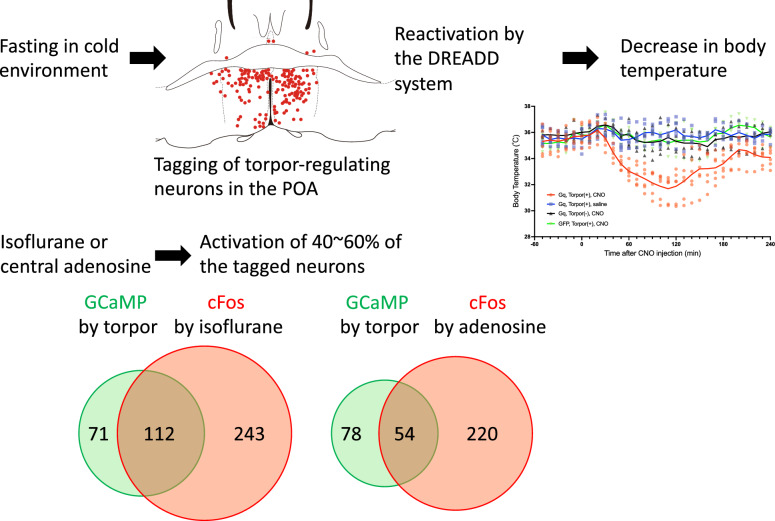

## Background

Some animals, especially tiny mammals living in cold climates, hibernate during winter. Although laboratory mice do not hibernate, they undergo daily torpor, a mild form of hibernation, to endure cold environments and fasting [1, 2]. During fasting and cold environment-induced daily torpor, body temperature decreases (to ~ 30 °C) cyclically several times in mice [2, 3]. The hypothermic episode starts ~ 10 h from fasting, lasts 1–2 h, and repeats 3–4 times during 24 h of fasting [3]. It is considered that hibernation and torpor are not passive processes caused by external temperature drops and fasting but are active brain functions that lower body temperature. The hypothalamic preoptic area (POA) has been regarded as the hibernation-regulating brain area since a study in hibernating squirrels showed activation of the deep brain, including the POA, but not the cerebral cortex [4]. The torpor-regulating brain area has scarcely been reported, although it was thought to be the POA from the analogy of hibernation and torpor.

A population of neurons in the POA was recently identified as fasting-induced active hypothermia-inducing neurons [5]. They are located in the medial and lateral POA of the hypothalamus and express vesicular glutamate transporter-2 and pituitary adenylate cyclase-activating polypeptide (PACAP) [5]. Another group found that activation of pyroglutamylated RFamide peptide (QRFP) expressing neurons in the anteroventral perivascular nucleus and medial preoptic nucleus resulted in long-lasting hypothermia and hypometabolism even without fasting euthermic condition and termed them as quiescence-inducing neuron (Q-neurons) [6]. The third group found that neurons expressing estrogen α-receptors in the medial POA are required for fasting-induced torpor [7]. Although whether those neurons are identical or not has not yet been confirmed, these reports convincingly showed the existence of hypothermia-inducing neurons in the medial POA.

Importantly, hypothermia-inducing mechanisms and restoring mechanisms may be different: we showed in orexin neuron-ablated mice a longer lasting and more profound hypothermia than in the wild-type mice and activation of orexin neurons in the wild-type mice during the recovery period, indicating a contribution of the orexin neurons in the recovery from hypothermia [3]. Hypothermia-inducing mechanisms were not known at that time. Therefore, in this study, we tried to identify hypothermia-inducing neurons by collecting the brain just after the nadir of the first episode of fasting-induced hypothermia and examining the active neuronal population using c-Fos immunoreactivity.

Other than hibernation and torpor, some maneuvers decrease body temperature and oxygen consumption. Of these, we focused on isoflurane anesthesia and central injection of adenosine. The former is of interest because of clinical implications. Although the anesthetics, including isoflurane, are known to reduce body temperature [8, 9] with decreasing vasoconstriction and shivering thresholds [10], precise mechanisms are poorly understood. Adenosine is a presumed metabolic signal of short energy supply into the brain, and both central and peripheral injections of adenosine or its analogs induce hypothermia [3, 11, 12]. We previously showed that hypothalamic orexin neurons play a protective role against hypothermia caused by isoflurane [8] and central administration of an adenosine A1 receptor agonist, N6-cyclohexyl adenosine (CHA) [3] since orexin neuron-ablated mice showed exaggerated hypothermia induced by isoflurane and CHA. However, the mechanism by which isoflurane and CHA induce hypothermia remains unclear.

## Methods

### Animals and ethics approval

All experimental procedures were performed following the guiding principles for the care and use of animals in the field of physiological sciences published by the Physiological Society of Japan (2015) and approved by the Institutional Animal Use Committees at Kagoshima University (MD17105).

For examining torpor-activated brain regions and the euthermic experiment (see below), we used male wild-type mice of C57BL/6 strain (Japan Clea) that were 4–6 months old and weighed 24–36 g. These mice were housed in a room maintained at 22–24 °C with lights on at 19:00 and off at 7:00 for at least 1 month before experimentation started. We selected a reversed light/dark cycle so experimenters could observe mice in their active phase during the daytime.

For an experiment on the reactivation of torpor-activated neurons, we used male TetTag mice (B6.Cg-Tg(Fos-tTA, Fos-EGFP*)1Mmay/J, Jackson Laboratory, Stock#018306). These mice were housed in a room maintained at 22–24 °C with lights on at 07:00 and off at 19:00. Mice had food and water ad libitum and were socially housed until the beginning of the surgery. The mice were 4–6 months old at the time of surgery and had been raised on food containing 200 mg/kg doxycycline and tap water retaining 700 mg/l doxycycline for 4 weeks before surgery. Mice were single-housed post-surgery and throughout the rest of the experiments.

Fos-tTA; TetO-GCaMP6 mice were used to examine whether torpor-activated neurons are also activated by other hypothermia-inducing maneuvers (isoflurane anesthesia or a central injection of adenosine receptor agonist). The mutant was generated by crossing TetTag mice with *TetO-GCaMP6* knock-in mice (*B6;129-Actb *<*tm3.1(tetO-GCaMP6)Kftnk*>, obtained from the RIKEN BioResource Research Center, RBRC09552). The double-transgenic mice were confirmed via PCR. We have demonstrated the usefulness of this mutant to examine the possible overlap of the different c-Fos inducing activation onto one neuron [13].

### Measurement of body temperature during fasting-induced hypothermia

Using a telemetric system (Dataquest, Data Sciences International, St Paul, MN, USA), we measured abdominal temperature in freely moving mice in their home cages. At least 7 days before the experiment, a telemetric device (TA11TA-F10, Data Sciences International) was implanted in the abdominal cavities of mice, while they were under anesthesia with 2–3% isoflurane. An antibiotic (penicillin, 40,000 U/kg) was subcutaneously injected. At 7:00, the beginning of the lights-off period, the mice were deprived of food in a mild cold environment at 21 °C to induce torpor, and the body temperature measurement was started. Water was freely available. A pyro-electric passive infrared sensor was attached to the ceiling of the home cage to detect the animal’s locomotor activity. The sensitivity of the movement sensor was set so that shivering would not be counted. Torpor was defined by a body temperature below 34 °C and decreased movement. Body temperature was continuously monitored and digitized by PowerLab (ADInstruments, Castle Hill, Australia) and was stored and analyzed using LabChart software (ADInstruments).

The core body temperature of Fos-tTA; TetO-GCaMP6 mice was intermittently measured using a wireless passive transponder system (Electric Laboratory Animal Monitoring System, BioMedic Data Systems, Inc., Seaford, DE, USA). Since the transponder (IPTT-300) is more miniature (14 mm × 2 mm × 2 mm vs. 20 mm × 12 mm × 5 mm) and lighter (0.12 g vs. 1.6 g) than the telemetric device (TA11TA-F10), it may be more suitable for animal husbandry. However, continuous measurement is problematic because, at every measuring time, the experimenter must switch on and hold the system’s scanner (DAS7007R) within 5 cm of the transponder.

### Stereotaxic injection AAV into POA

Surgeries for injections were performed under isoflurane (1.5%, inhalation) anesthesia using a stereotaxic instrument (ST-7, Narishige, Tokyo, Japan). A glass micropipette (2–000-001, Drummond Scientific, Broomall, PA, USA) pulled with a puller (PC-10, Narishige) with a tip diameter of 10 μm was filled with recombinant adeno-associated virus (AAV-DJ-pTRE-tight-hM3Dq-mCherry (Addgene Plasmid #66795 packaged by Dr. Kenta Kobayashi at National Institute for Physiological Sciences, 1.2 × 10^10^ GC/ml) or AAV-DJ-tetO-EGFP (a kind gift from Prof. Akihiro Yamanaka at Nagoya University, 9 × 10^9^ GC/ml). AAVs (60 nl/side during 10 min) were stereotaxically and bilaterally injected into the POA of TetTag mice. A gas pressure injector system (IM-200J, Narishige) was connected to the glass micropipette and a compressed nitrogen tank with a polyethylene tube. A pressure (5–10 kPa) was applied to inject the solution. Injection sites were anterior 0.5 mm, lateral ± 0.5 mm, and ventral 5.4 mm from bregma. After pressure injection, the micropipette was left in place for 10 min before being slowly withdrawn. At the same time, a telemetric device was also implanted in the abdominal cavities of mice. After surgery, mice were given an antibiotic (penicillin G, 40,000 U/kg) and an analgesic (buprenorphine, 0.05 mg/kg). Mice were allowed to recover for more than 2 weeks before the experiment.

### Activity tagging with fasting-induced hypothermia

Activity tagging techniques used in this study involve the tetracycline-dependent gene control system [14, 15]. The transgenic mouse cells carrying the *c-fos* promoter-driven tetracycline transactivator (tTA) gene will express tTA only when c-Fos activating signals come. tTA binds the tetracycline response element (TRE), and transcription of the object gene (in this study, hM3Dq or GCaMP6) will begin. Doxycycline, a potent tetracycline analog, can prevent the binding of tTA and TRE—removal of doxycycline from the food and drinking water switch on the system to run. We used fasting as the c-Fos activating signal instead of giving doxycycline-free rodent chaw.

Mice were maintained with food containing 200 mg/kg of doxycycline and tap water holding 700 mg/kg of doxycycline. For activity tagging, doxycycline was replaced with doxycycline-free food and water 24 h before the fasting. A paper showed that the half-life of the doxycycline in the mouse was 170 min [16]. Therefore, 99.7% of doxycycline will disappear in 1 day. The activity-tagging stimulus was started at ZT12, the beginning of the light-off time. The mice were deprived of food in a mild cold environment at 21 °C, and the body temperature measurements were started. Doxycycline-free water was freely available. After 12 h and confirmation of torpor induction, the food and water were replaced with that containing doxycycline. After 48 h from the fasting stimulation, the clozapine *N*-oxide (CNO) experiment was started. Mice were given i.p. injection of CNO (0.045 mg/kg) or vehicle at 4:00 (around the time when the fasting-induced hypothermia began to occur).

### Overlapping experiment

To examine whether other hypothermia-inducing maneuvers also activate torpor-activated neurons, we first tagged torpor-activated neurons in the Fos-tTA; TetO-GCaMP6 mice in the same way as in AAV-injected Fos-tTA mice (see above activity-tagging section). After 48 h from the fasting stimulation, the animals received one of the following treatments. (1) The isoflurane group received isoflurane anesthesia (1.5% in the room air) for 90 min. (2) The air control group was confined to the anesthesia chamber without isoflurane (airflow 0.6 l/min). (3) Intracerebroventricular administration of an adenosine A1 receptor agonist, N6-cyclohexyl adenosine (CHA; Sigma-Aldrich). Stock solution of CHA (5 mM in saline) was diluted with artificial cerebrospinal fluid (ACSF) to be 20 µM. According to our previous report [3], the dose was selected. 4) Intracerebroventricular administration of ACSF (2 µl/mouse) as the vehicle control. For groups 1) and 2), mice were acclimatized to the anesthesia chamber three times before the experiment. For groups 3) and 4), a guide cannula (C315GS-5/2.5; Plastics One Inc., Roanoke, VA, USA) for the intracerebroventricular administration of drugs to the lateral ventricle (1 mm lateral to the Bregma, 2.5 mm deep in the skull) was implanted at least 7 days before the experiment. They received CHA or ACSF in their home cage. No torpor-tagged animals also received one of the above four treatments as the control.

### Euthermic experiment

We next examined whether isoflurane or CHA activated the POA neurons through their pharmacologic effect or the resulting hypothermia by administering isoflurane or CHA to the wild-type mice while keeping their body temperature. The wild-type mice indwelling a wireless passive transponder (IPTT-300, BioMedic Data Systems, Inc., Seaford, DE, USA) were administered 1.5% isoflurane or 0.04 nmol (20 µM × 2 µL) of CHA. At the same time, the anesthesia chamber or home cage was immersed in the water bath (37 ± 1 °C) to keep the animals’ body temperature euthermic [8]. The control groups received room air or ACSF administration.

### Immunohistochemistry

**Fig. 1:**
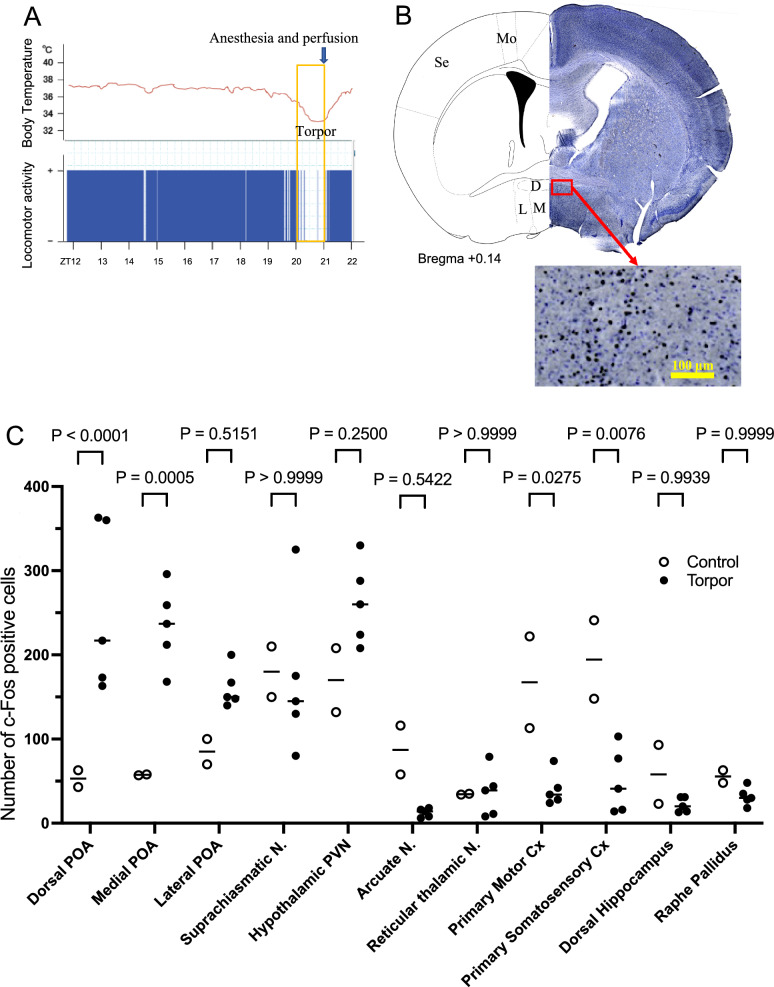
Distribution of torpor-inducing neurons in the brain. **A** Schematic protocol of the experiment. A telemeter-indwelling mouse in its home cage was put into a chilled (21 °C) environment without food from zeitgeber time (ZT) 12 while measuring body temperature and locomotor activity. Induction of torpor was defined as the decrease in body temperature and disappearance of locomotor activity. When the body temperature began to rise again after the nadir of hypothermia, the animal was quickly anesthetized with i.p. injection of urethane, and intracardiac perfusion was started for brain sampling. **B** Typical example of a mouse’s brain showing c-Fos-positive neurons in the preoptic area (POA). D dorsal POA; L, lateral POA; M, medial POA; Mo, motor cortex; Se, somatosensory cortex. **C** The number of c-Fos positive neurons in the 11 brain regions from 5 torpor-experienced and 2 control mice. Two-way ANOVA showed there is a significant difference among brain regions (F (10, 55) = 9.338, *P* < 0.0001) and interaction (brain regions x torpor condition) (F (10, 55) = 8.017, *P* < 0.0001) but not between condition (F (1, 55) = 1.011, *P* = 0.3191). *P* values on the top are the pairwise difference probability between torpor conditions in each brain region, calculated by Sidak’s multiple comparison test. Abbreviations: PVN, paraventricular nucleus; Cx, cortex

For endogenous c-Fos staining following fasting-induced hypothermia, mice were euthanized and perfused after 10 min when the body temperature began to rise again after the nadir of hypothermia. Since the onset and depth of fasting-induced hypothermia vary among the mice and body temperature sometimes changes stepwise rather than smooth [3], we continuously measured the body temperature and collected the sample appropriately (see Fig. 1A). We selected the sampling time to be 10 min after the starting point of the recovering phase for two reasons: first, to confirm the end of the nadir of the torpor, we thought the 10 min of waiting time was needed. The second reason was that we used c-Fos immunohistochemistry to examine possible neuronal activation. Production of c-Fos protein is the largest after 30–90 min of the stimulation [17], and the fasting-induced torpor in our experimental condition lasts in a similar time range. Therefore, we waited for all the falling and nadir phases to obtain maximum c-Fos expression. As a control, we perfused non-fasted mice at ZT21, almost the same period as the experimental group.

For c-Fos staining and detecting activity-tagged hM3Dq-mCherry expression, mice were euthanized and perfused 4 h after the injections of CNO. For overlapping experiments and the euthermic experiments, mice were euthanized at the end of isoflurane exposure or 90 min after the infusion of CHA.

Mice were deeply anesthetized with urethane (1.8 g/kg, i.p.) and transcardially perfused with 25 ml of phosphate-buffered saline (PBS, 0.01 M, pH 7.4), followed by 25 ml of 4% paraformaldehyde (PFA) in PBS. The brain was removed, post-fixed in 4% PFA solution at 4 °C overnight, and immersed in 30% sucrose in PBS at 4 °C for 2 days. A series of 40 μm sections were obtained with a vibratome (SuperMicroSlicer Zero1; DOSAKA EM, Kyoto, Japan), of which every fourth section was used for immunostaining. The brain sections were immersed in a blocking solution (1% normal horse serum and 0.3% Triton-X in 0.01 M PBS) for 30 min at room temperature. The sections were then incubated with a primary antibody that was an anti-c-Fos rabbit antibody (ABE457, Millipore Corp., RRID: AB_2631318, 1/1000) for 90 min. Primary antibodies were diluted in a blocking solution. The sections were washed with PBS and then incubated with an anti-rabbit IgG-biotin complex secondary antibody (711-065-152, Jackson ImmunoResearch, RRID: AB_2340593, 1/300) for 2 h. The sections were washed with PBS and then incubated with an ABC system (PK-7100, Vector Laboratories, RRID: AB_2336827) for 90 min and visualized with DAB staining (SK-4100, Vector, RRID: AB_2336382) with cobalt and nickel intensification. Finally, sections were counterstained with Cresyl violet (conventional Nissl staining). The number of c-Fos-positive cells was counted in 11 brain regions reportedly involved in hibernation [4].

For c-Fos staining in AAV experiments, the fluorescence method was used instead of the above conventional method because the AAV used also has the fluorescent marker mCherry or GFP. After blocking the solution, the sections were incubated with anti-c-Fos rabbit antibody (2250(9F6), Cell Signaling Technology, RRID: AB_2247211, 1/1000) for 1 night. The sections were washed with PBS, and c-Fos was visualized with CF488-labeled anti-rabbit IgG raised in donkeys (Biotium 20015, RRID: AB_10559669, 1/500).

For the overlapping experiment, the mice were transcardially perfused with Ringer’s solution (containing 3 mM CaCl_2_), followed by 4% paraformaldehyde in 0.1 M Tris (pH 7.4) + 3 mM CaCl_2_. We added calcium to the ordinal washing and fixative solutions because we found in our preliminary experiment that the fluorescence of GCaMP6 was better preserved in supplementation with calcium [13]. Expression of c-Fos induced by isoflurane or CHA was visualized using anti-c-Fos rabbit antibody (2250(9F6), Cell Signaling Technology, RRID: AB_2247211, 1/1000) and CF568-labeled anti-rabbit IgG raised in donkey (Biotium 20098, RRID: AB_10853318, 1/500). Expression of GCaMP6 and c-Fos-immunoreactivity was observed by epi-fluorescent microscope (BZ700, Keyence, Osaka, Japan) and counted in the medial and dorsal POA area.

### Statistical analysis

Statistical analyses were performed via paired t-test or one- or two-way ANOVA with post hoc Sidak’s multiple comparisons test using the Prism10 software (GraphPad Software, San Diego, CA, USA.). Statistical significance was set at *P* < 0.05 in all analyses.

## Results

### Torpor activates the neurons in the preoptic area

To identify the brain regions activated during the entering phase of torpor, we systematically surveyed c-Fos expression in the brain. The mice were euthanized and perfused when the body temperature began to rise again after the nadir of hypothermia (Fig. 1). Although hypothermia repeats several times during the fasting period [3], we used only the first hypothermic event to avoid possible carryover effect from the preceding hypothermia and recovery. As a control, we perfused non-fasted mice at ZT21, almost the same period as the torpor group. Of 11 brain regions so far examined, a significant difference in c-Fos expression was observed in 4 areas between the torpor and control groups (Fig. 1C). Among those 4 regions, dorsal and medial preoptic areas (POA, Fig. 1B, and C) were the only region that showed higher expression of c-Fos during torpor than the control. Motor and somatosensory cortex showed significantly lower c-Fos expression during torpor. This finding suggests that activation of the neurons in POA might induce hypothermia to enter a fasting-induced torpor state.

### Reactivation of torpor-active neurons using the DREADD system

**Fig. 2:**
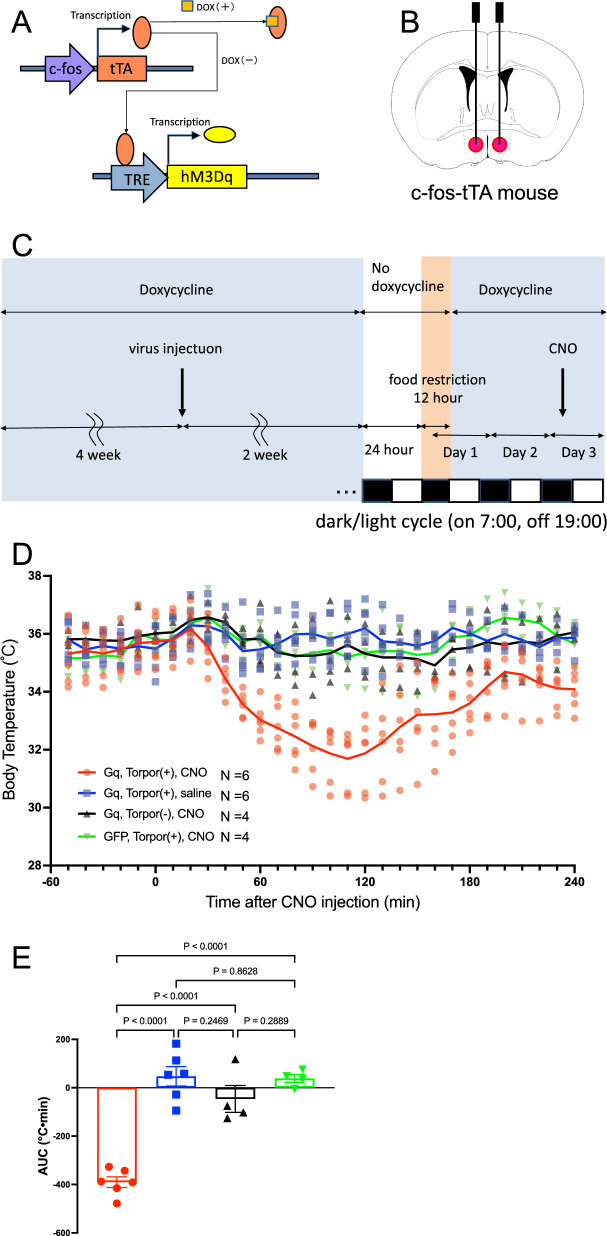
Reactivation of the torpor-tagged neurons. A–C Activity tagging strategy used in this study. **A** Schematic explanation of the doxycycline (Dox) off switch. **B** Bilateral injection of AAV-DJ-pTRE-tight-hM3Dq-mCherry into the POA of Fos-tTA mice. **C**. Timeline of the experiment. **D** Time course of the body temperature changes in the mice received CNO or saline at the time 0. Only the torpor-tagged Gq-expressing mice that received CNO showed an apparent decrease in body temperature. The number of animals used in this experiment was 6 for Gq., torpor ( +), CNO; 6 for Gq., torpor (+), saline; 4 for Gq., torpor (-), CNO; 4 for GFP, torpor ( +), CNO. **E** The lowering effect of body temperature was evaluated as the area under the curve (AUC) below the pre (from -60 to 0 min) value from 0 to 180 min after the drug injection. One-way ANOVA showed a significant difference among the groups (F (3, 16) = 37.25, *P* < 0.0001). *P* values on the top are calculated using Sidak’s multiple comparison test. The error bar indicates S.E.M

To determine whether activation of the neurons in POA plays a causative role in hypothermia, we took a genetic strategy to express Gq-coupled designer receptor exclusively activated by designer drug (DREADD) specifically in the POA neurons that are active when mice enter torpor (Fig. 2A–C). AAV-DJ-pTRE-tight-hM3Dq-mCherry was injected into the POA of TetTag mice, which express tTA under the control of immediate early gene *c-fos* promoter. Infected cells produce hM3Dq only when *c-fos* is activated without the antibiotic doxycycline (dox) in diet and drinking water [14, 15]. Targeted neurons expressing hM3Dq can be reactivated later by administering DREADD-activating synthetic ligand CNO without fasting stimulus. We call TetTag mice infected with AAV- hM3Dq in their POA “TetTag-Gq” mice.

Two weeks after the injection of AAV into POA, TetTag-Gq mice (*n* = 6) were fed with no dox diet and water for 24 h and then fasted for 12 h. After 2 days of recovery from fasting, they were administered with CNO to reactivate the POA neurons that were previously activated by natural torpor (Fig. 2C). We found an intense and long-lasting reduction of core body temperature and locomotion by reactivation with CNO of tagged cells without fasting (Fig. 2D, E). Administration of saline (*n* = 5) did not affect body temperature. Tagging of the fasting-induced torpor-activated cells seems successful because an administration of CNO into TetTag-Gq mice (*n* = 4) without torpor experience did not result in a torpor-like decrease in body temperature. In addition, administration of CNO did not change body temperature in TetTag-GFP mice (*n* = 4), which received AAV-DJ-tetO-EGFP instead of AAV-DJ-pTRE-tight-hM3Dq-mCherry in their POA and experienced tagging stimulus of fasting. Although we cannot exclude the possible contribution of neurons other than those in POA to fasting-induced torpor, our finding shows that the activation of POA neurons is sufficient to reduce core body temperature during torpor.

### Distribution of torpor-tagged and CNO-activated cells in the POA

**Fig. 3:**
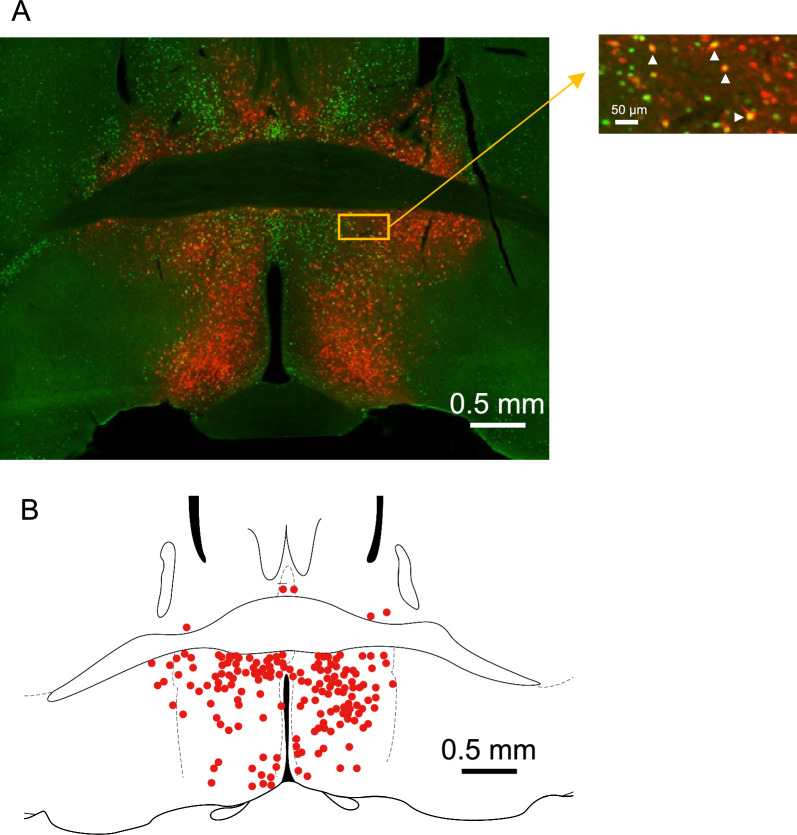
Distribution of the torpor-tagged and CNO-reactivated neurons in the preoptic area. **A** A typical photograph was taken from a TetTag-Gq mouse that experienced torpor and CNO injection. White rectangles in the enlarged photograph show double-stained cells for Gq-mCherry (red) and c-Fos (green). **B** Schematic diagram showing the distribution of double-stained cells from 6 mice

Although AAV infected many neurons in the POA, torpor-mediating neurons might be a part of them. To examine the issue, we collected the TetTag-Gq brain after 4 h of body temperature measurement, and c-Fos distribution was analyzed by fluorescent immunohistochemistry. As expected, many neurons in the POA were simultaneously labeled with Gq (mCherry) and c-Fos (green) (Fig. 3A). Double staining indicates that these neurons were labeled by preceding torpor and reactivated by CNO. Double-labeled neurons were mainly distributed in the medial preoptic and dorsal preoptic nuclei (Fig. 3B).

### Overlapping of torpor-tagged neurons and isoflurane- or adenosine-activated neurons

**Fig. 4:**
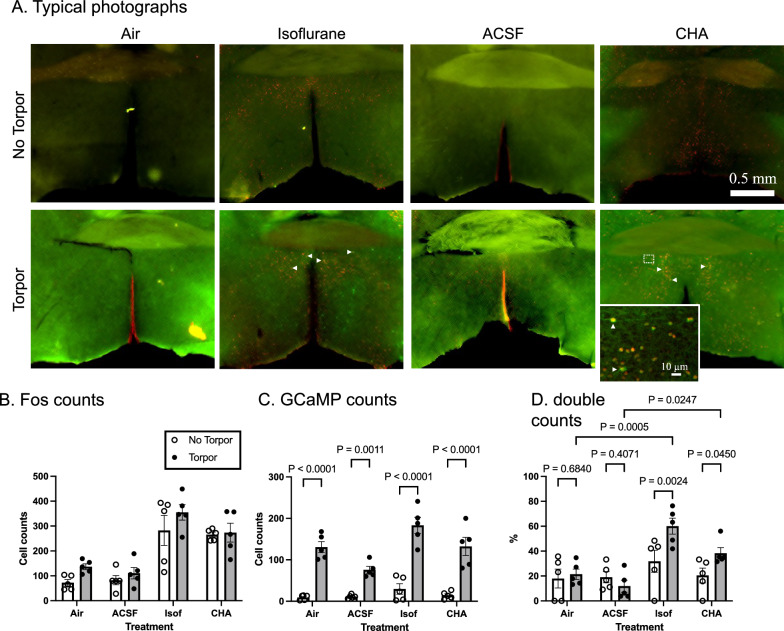
Overlapping of torpor-tagged neurons and isoflurane- or adenosine-activated neurons. **A** Typical photographs show torpor-experienced (lower panels) and un-experienced (upper panels) TetTag-GCaMP mice brains. They received air, isoflurane, ACSF, or CHA-treatment 48 h after the fasting torpor. GCaMP is shown in green, and c-Fos immunoreactivity is visualized in red. A white rectangle indicates some of the double-stained cells. The inset shows an enlarged photograph in the white dotted square. **B** The number of c-Fos-immunoreactive cells increased by isoflurane- and CHA-treatment irrespective of the preceding torpor: effect of torpor, F (1,32) = 4.127, *P* = 0.0506; effect of treatment, F (3, 32) = 28.28, *P* < 0.0001; interaction, F (3, 32) = 0.5115, *P* = 0.6772; 2-way ANOVA, *n* = 5 for each group. The error bar indicates S.E.M. **C** The number of GCaMP positive cells increased by torpor: effect of torpor, F (1,32) = 161.3, *P* < 0.0001; effect of treatment, F (3, 32) = 8.132, *P* = 0.0004; interaction, F (3, 32) = 4.175, *P* = 0.0133; 2-way ANOVA. As indicated in the figure, the *P *value within a treatment was calculated using Sidak’s multiple comparison tests. **D** The percentage (GCaMP and Fos/GCaMP) of double-positive cells increased by isoflurane- and CHA-treatment in a torpor-dependent manner: effect of torpor, F (1,32) = 47.69, *P* < 0.0001; effect of treatment, F (3, 32) = 14.71, *P* < 0.0001; interaction, F (3, 32) = 9.584, *P* = 0.0001; 2-way ANOVA. The *P* value between the groups was calculated using Sidak’s multiple comparison tests, which are indicated in the figure

To examine whether other hypothermia-inducing maneuvers also activate torpor-activated neurons, we used TetTag-GCaMP mouse and counted GCaMP and c-Fos expression in the POA (Fig. 4). Isoflurane induced hypothermia (from 36.1 ± 0.4 to 30.1 ± 0.6 °C, *n* = 10, *P* = 0.0001, paired t-test) and CHA induced hypothermia (from 35.8 ± 0.5 to 28.5 ± 1.3 °C, *n* = 10, *P* = 0.0002, paired *t*-test). Both maneuvers increased c-Fos expression in the POA, irrespective of preceding torpor (Fig. 4B). As expected, GCaMP count was dependent on preceding torpor (F (1,32) = 161.3, *P* < 0.0001, two-way ANOVA) (Fig. 4C). The percentage of double (GCaMP and c-Fos) labeled neurons in the torpor-tagged neurons was significantly more prominent in the torpor and isoflurane condition (60.0 ± 6.4%) than in torpor and air condition (21.4 ± 4.1%, P = 0.0024) (Fig. 4D). That in the torpor and CHA condition (38.3 ± 4.5%) was also larger than torpor and ACSF condition (11.8 ± 4.8%, *P* = 0.045). These results showed that torpor and isoflurane activated some of the same neurons. The same was valid for torpor and CHA.

### Isoflurane and adenosine activate POA neurons without hypothermia

**Fig. 5:**
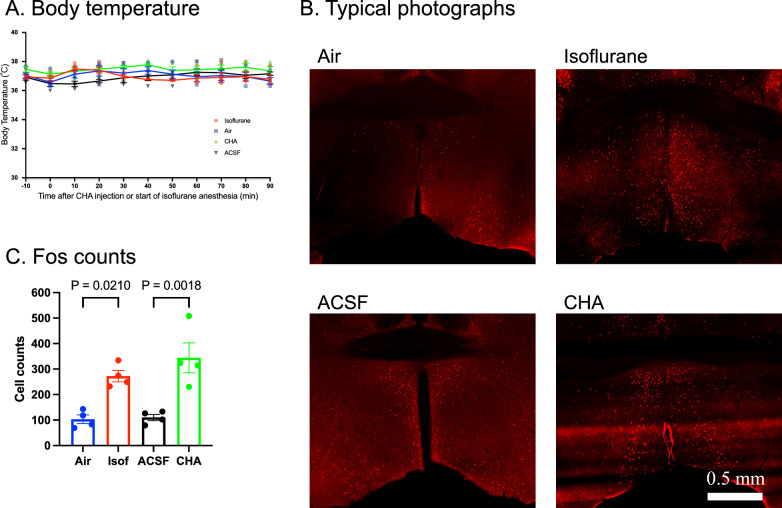
Isoflurane and adenosine activate POA neurons without hypothermia. **A** Putting the anesthesia chamber (in the isoflurane or air exposure) or the mouse home cage (in the CHA or ACSF administration) in a warm (37 °C) water bath successfully kept the body temperature: effect of time, F (3.911, 46.93) = 2.146, *P* = 0.0912; effect of treatment, F (3, 12) = 3.059, *P* = 0.1593; interaction, F (30, 120) = 1.730, *P* = 0.0202; 2-way ANOVA (repeated measure, Geisser–Greenhouse correction). **B** Typical photographs show the mice brains received air, isoflurane, ACSF, or CHA in the euthermic condition. The immunoreactivity of c-Fos is visualized in red. C. The number of c-Fos-immunoreactive cells increased by isoflurane and CHA-treatment in the euthermic condition: effect of treatment, F (3, 12) = 13.20, *P* = 0.0004; 1-way ANOVA, *n* = 4 for each group. The *P *value between the groups was calculated using Sidak’s multiple comparison tests, which are indicated in the figure. The error bar indicates S.E.M

Mice were successfully kept euthermia during isoflurane anesthesia and after the injection of CHA by warming their cages in a 37 °C water bath (Fig. 5A). Nevertheless, isoflurane and CHA increased c-Fos expression in the POA as compared to their respective controls (Fig. 5B, C). The effect of isoflurane and CHA on the number of c-Fos-positive cells in the POA was similar to those in hypothermic condition (~ 300 cells, compare Fig. 5C and Fig. 4B). These results indicated that the increase in c-Fos in the POA was caused by the pharmacologic effect of isoflurane and CHA but not by the indirect impact from resulting hypothermia.

## Discussion

This study confirmed that torpor induction was associated with POA neuronal activation. We showed that reactivation of the previously torpor-tagged GqDREADD-expressing neurons in the POA induced long-lasting hypothermia. We also showed that isoflurane and CHA activated about 40–60% of torpor-tagged neurons. Isoflurane and CHA activated the POA neurons even when the body temperature kept euthermic. Isoflurane-induced and central adenosine-induced hypothermia is, at least in part, an active process mediated by the torpor-regulating neurons in the POA.

Compared to previous pioneering studies reporting torpor-regulating neurons [5] and Q-neurons [6], this study showed generally similar results in the context of hypothermia-inducing neurons in the POA. However, the location of hypothermia-inducing neurons is slightly different: Hrvatin et al. [5] reported medial and lateral POA, Takahashi et al. [6] said the anteroventral perivascular nucleus and medial preoptic nucleus, and this study indicated medial and dorsal POA. All the reports showed the involvement of medical POA; thus, common features of the neurons involved in the induction of hypothermia should be located there. Different distributions other than the medial POA indicate subpopulations of the hypothermia-inducing neurons. Our tagging system (TetTag) differs from the previous report, in which the authors used a cre-loxP system with an activation switch of 4-hydroxy tamoxifen [5]. Nevertheless, a similar hypothermia-inducing effect was observed by reactivating the torpor-tagged neurons, indicating the validity of both tagging methods.

Although we tried to label only torpor-entering neurons in our first experiment (Fig. 1), we cannot distinguish between torpor-entering neurons and recovery neurons in our tagging experiment since the time window for tagging included both torpor-entering period and recovering period (Fig. 2C). However, reactivation of the tagged neurons resulted in torpor-like hypothermia but not hyperthermia (Fig. 2D, E), indicating they were torpor-entering neurons and torpor-recovering neurons were located in elsewhere than the POA. Another possibility was that the torpor-entering neuronal activity predominated over torpor-recovering neuronal activity even if both neurons were in the POA.

This study showed the possible contribution of the torpor-tagged neurons in isoflurane-induced hypothermia and central CHA-induced hypothermia. Hrvatin et al. [5] showed that peripheral injection of CHA did not affect the activity of their torpor-regulating neurons. Our observation did not conflict with their results because adenosine receptors are distributed in the brain and peripheral organs, and the dose to induce hypothermia was 100 times more potent in the central injection [3, 11, 12]. It is plausible that several maneuvers activate a common neuronal population to decrease body temperature.

We are aware of the limitations of this study. We only showed the possibility of isoflurane- and CHA-induced hypothermia depends on torpor-tagged neurons. Further experiments are needed to draw conclusive results, such as torpor-tagged neuronal inactivation and measuring vasoconstriction and shivering thresholds.

In sum, this study confirmed a torpor-regulating active mechanism in the POA. Torpor-regulating neurons in the POA may also serve for isoflurane- and central adenosine-induced hypothermia. Our method opens the way to searching for humane methods to actively lower the body temperature in medical settings, such as reducing brain damage after cardiac arrest and brain injury [18].

## Data Availability

The article contains summary statistics. The raw data supporting this study’s findings are available from the corresponding author upon reasonable request.
